# Towards an effective collaboration between the South Western Sydney Local Health District and local councils: insights from a qualitative study

**DOI:** 10.1186/s12961-022-00850-1

**Published:** 2022-04-28

**Authors:** Vilas Kovai, Zeenat Mahjabeen, Bin Jalaludin, Francis Fox

**Affiliations:** 1 Population Health-Health Promotion Service, South Western Sydney Local Health District, Waranara Building, Eastern Campus, Liverpool Hospital, Liverpool BC, NSW 1871 Australia; 2 School of Social Sciences, Faculty of Arts, Architecture and Design, The University of New South Wales, Kensington, NSW 2052 Australia; 3 Population Health Intelligence, Health People and Places Unit, South Western Sydney Local Health District Locked, Bag 7279, Liverpool, NSW 1871 Australia

**Keywords:** Built environment, Collaboration, Councils, Evidence-based local data, Engagement, Population health, Policy, Qualitative research, Urban planning

## Abstract

**Background:**

Partnership between local government and local health districts is imperative, given their overlapping goals. However, the need for further evidence-informed actions to address health inequities remains. The effectiveness of such partnerships requires better insight into how local governments perceive partnerships with local health districts, and how well equipped and prepared they are to deal with the health equity opportunities and challenges. It was precisely for these reasons that a qualitative study was conducted by South Western Sydney Local Health District (SWSLHD) in 2016.

**Objective:**

This study aims to better understand how to improve the effectiveness of collaboration between local governments and the public health sector.

**Methods:**

Qualitative data were collected from 14 in-depth interviews with staff representing five of the local councils comprising SWSLHD. These data were then thematically analysed using inductive and deductive reasoning through the application of NVivo software.

**Results:**

While councils recognize the potential value of consulting SWSLHD, limited communication and the absence of a clearly defined process for collaboration needs to be addressed. Moreover, councils perceive knowledge gaps in relation to basic issues, such as who provides what services to whom, and how to access local-government-level data from health experts.

**Conclusions:**

The study confirms the importance of providing locally relevant public health data to help address issues of mutual concern that arise during the consultation process. Moreover, it suggests that proactive and ongoing consultation between SWSLHD and councils is critical if there is to be effective engagement, and coordinated and sustained action. The concerns raised in this study echo findings from studies from other local government settings of Victoria, South Australia and New South Wales. Thus, the study findings may be applied to other councils beyond the SWSLHD.

## Background

Health inequities exist between countries, within countries and between neighbourhoods [1], and are determined by factors ranging from the individual to the social-structural [1, 2]. Biological and behavioural factors at an individual level are amenable to healthcare and behavioural or downstream interventions. Health inequities determined by social structural factors, however, can be addressed by structural or upstream interventions, such as changes to the built environment [3].

Empirical evidence confirms the association between features of the built environment—such as neighbourhood design, access to green spaces, amenities and facilities, healthy food environment, transport, affordable housing—and health and well-being [3]. Neighbourhood features such as enhanced street connectivity, lighting and land-use mix were associated with improved physical activity [4] and social engagement in the general population [3–5]. Similarly, access to infrastructure facilities for walking, cycling and public transportation are associated with increased mobility and resultant cardiovascular benefits in the general population [4]. Living in neighbourhoods with green spaces is more likely to reduce the risk of mortality from cardiovascular disease [6]. Access to facilities and amenities were associated with increased social participation and improved mental health in older adults [7].

It is important to note that much of the above evidence comes from cross-sectional studies, though a causal relationship between the built environment and health impacts and outcomes is difficult to determine through randomized control trials, as these studies are not feasible in complex community settings [3–5, 8]. A recent systematic review [3], for example, suggests that further evidence from quasi-experimental studies is required to establish the effectiveness of interventions related to built environment and health outcomes.

Given the need for further evidence-informed action to address health inequities [1], particularly from robust quasi-experimental studies and prospective controlled studies [3, 9], it is imperative to establish a strong collaborative partnership between public health professionals and local government. This partnership is particularly important, as it is local government that plans and implements programmes or services related to neighbourhood design, provision of services enabling access to healthy food, physical activity and smoking cessation, and the reduction of crime related to alcohol and gambling behaviours [10].

Globally, local government has been recognized for its role in protecting and promoting health [11–13]. WHO [14] and its commission on social determinants of health [15] has recognized the importance of local government’s role with respect to addressing social determinants of health and health equity, and there is a strong call on local governments worldwide to play a leading part.

In Australia, both federal and state governments have encouraged the role of local governments in addressing the upstream social determinants of health [13]. For example, in Australia, the Government of Victoria’s Municipal Public Health Planning Framework [16], puts emphasis on the public health needs of local communities and local leadership in promoting health and well-being through built, social, economic and natural environments. In the state of New South Wales (NSW), a Healthy Built Environment Checklist was developed to ensure effective engagement between health and planning professionals and support the development of well-connected, liveable and sustainable communities [17].

While the upstream determinants of health such as built environment and local economic development and housing [18] could be well planned and delivered by local government authorities [19], currently, many local governments in Australia still have a regulatory public health role to look into the food safety, water and air quality in their jurisdictions and contribute to protecting the health of their communities [11]. These activities of local government are important; however, much more beyond this needs to be in place to address the major upstream social determinants of health associated with noncommunicable diseases and other causes of morbidity and mortality [11, 19].

In Australia, local government areas (LGAs) act as a third tier of government, with local councils accountable for the planning, design and implementation of programmes related to the built environment of their communities [10]. Local government impacts the structural social determinants of health through its regulatory powers, community services, local leadership and contribution to the creation of a healthy environment [20]. In fact, it is the primary responsibility of local health districts to promote, protect and maintain the health of the community. They are also responsible for managing public hospitals and health institutions, and providing health services to defined geographical areas.

Hence, a coordinated and sustained action through partnership between councils and the South Western Sydney Local Health District (SWSLHD) and other sectors is crucial to maximizing the health outcomes for their communities. SWSLHD is one of 15 local health districts in NSW state. These local health districts were established as statutory corporations under the Health Services Act 1997 [21]. Of these, eight local health districts cover the Sydney metropolitan region and seven cover the rural and regional areas.

Currently, there is a dearth of evidence about how the local government staff perceive the need for partnership between the local government and health district, and existing opportunities and challenges to achieving more health equity gains [22, 23]. To gain better insight into the extent and effectiveness of partnership between councils and SWSLHD, a qualitative study was conducted in 2016 with staff involved in policy development at local councils. The study aimed to investigate how SWSLHD could be effectively involved in the urban planning process of local councils, and thereby promote a healthier urban environment. In this paper, the terms “local government” and “council” are used interchangeably for local government. Similarly the terms “local health district” and “SWSLHD” are used interchangeably for SWSLHD.

## Methods

This paper consists of an analysis of in-depth interviews with local council staff. The study participants were officers, managers and team leaders from various council departments and were working in areas directly related to policy, local environmental and community development. The participants were selected on the basis of their role in the councils and hence deemed to be suitable to answer the study questions. The purpose of using in-depth interviews in this study was two-fold: first, to obtain comprehensive information on the topic of interest, and second, to attempt to elicit additional information by asking follow-up questions. This study was approved by the Human Research Ethics Committee of the SWSLHD (HE 16/069 LNR). The consolidated criteria for reporting qualitative research (COREQ) [24] were used to report the important aspects of the study methodology in this paper.

The study was conducted in five of the seven LGAs in South Western Sydney, a region with a population of approximately 1 million, including the largest population of culturally and linguistically diverse (CALD) communities in NSW, with 36% of residents born overseas and about 49% of families speaking a language other than English at home [25]. Such demographic characteristics and a growing population with chronic and complex healthcare needs, including obesity and chronic disease [26], make this region an ideal location for our study.

In-depth interviews were conducted with staff from five of the SWSLHD’s seven local councils, namely Camden, Liverpool, Campbelltown, Canterbury-Bankstown and Fairfield. Wollondilly Shire Council and Wingecarribee Shire Council were excluded from the study for logistical reasons.

An open-ended tool to guide the interview process was developed through a literature review and discussion with the study team (Table 1). The topic guide for the interview included the following major themes: scope for SWSLHD’s involvement in the local councils’ built environment planning process and the barriers to and enablers of the effectiveness of that involvement.

**Table 1:** Open-ended guide for in-depth interviews

Domain	Open-ended question
Exploring the opportunities	What are the formal and informal opportunities for providing input? (Discussion about opportunities outlined in the environmental planning policies and any opportunities which are not outlined in the policies)
Using the opportunities	In what stages of the planning process is SWSLHD invited to provide input and what other health-related stakeholders are also invited to provide inputs?
Exploration and analysis of the factors of effective use of the opportunities	a. Providing/sharing information When SWSLHD is invited to provide input, what are the other things local councils provide them with the draft plan (like information package)? b. Perception in the local councils How important do the local councils think it is to consult SWSLHD before a plan is made? How the submissions are analysed and decided—what are the factors considered? c. Problems in accepting the inputs What are the problems local councils face in accepting/incorporating SWSLHD’s input into plans? How can they be addressed? If there is any, further discussion on the reasons d. Benefits/level of satisfaction What are the benefits the local councils think they are getting if SWSLHD is consulted? (May be increase in knowledge/skill, better relationship, and plan quality) a. Communication How comfortable are local councils to deal/communicate with the SWSLHD? Reasons for the opinion. What are the informal avenues of communication; if there are any, how often and whether that helps? b. Issues of accountability (transparency/access to information/integrity) Whether SWSLHD is provided with feedback after plans are finalized—such as what happens with their input, how much has been adopted or not and why
Towards healthier urban plans	Is the way SWSLHD provides input helpful or useful? If yes, how can be more improved? If no, why not? What are the steps or policy alternatives local councils think should be which can help them in planning for a better health-supportive urban environment

General managers of the nominated local councils were approached by a study investigator via phone and an email containing a letter from the Director of Population Health and a participant information sheet. The letter explained the purpose of the study and requested the participation of local council staff involved in the development of policy planning and programme implementation. This was then followed by a phone call from the principal investigator, seeking the willingness of councils to participate. Five councils responded within 2 weeks, expressing their willingness to participate and listing the contact details of potential interviewees. Study investigators then followed up by contacting each of the recommended staff members by email or phone, seeking an appointment.

Once interviewees had consented and indicated the times of their availability, appointments were made. Before each interview, informed consent was obtained. Investigators with prior experience in conducting qualitative studies conducted 14 face-to-face interviews at the council chambers. The duration of the interviews ranged from 45 to 60 minutes. All interviews were audio-taped and later transcribed verbatim by professional transcribers.

### Data analysis

The six steps of thematic analysis proposed by Braun and Clarke [27] were followed for data analysis. The data were coded using open and axial methods. One of the researchers (VK), in consultation with another researcher (ZM), developed an initial coding scheme using the topics of the interview guide and by familiarizing themselves with the transcripts. This coding framework consisted of themes, subthemes and their definitions. The initial coding framework was tested using a data set. Working independently, both researchers coded the data from three interviews and then evaluated the success of the coding process and findings. By doing so, they were able to enhance coding consistency and refine the coding framework, which was subsequently used for the data analysis of the 14 interviews. Finally, one researcher (VK) performed the data analysis using both inductive and deductive reasoning using NVivo version 10 software (QSR International, Doncaster, VIC, Australia) [28]. The open codes of data were merged into relevant categories, then into initial themes, and finally, the initial themes were reduced to a few major themes using the axial coding process.

## Results

### Participants

The results are based on an analysis of data from in-depth interviews with 14 participants from five of the seven local councils in SWSLHD (Table 2). The participants were all staff (officers, managers and team leaders) from various council departments, including Asset management for Open Space and Property (*n* = 1), Community Resources and Development (*n* = 1), Strategic Planning (*n* = 6), Recreation and Open Space Planner (*n* = 1), Spatial Planning (*n* = 1), Community Safety and Crime Prevention (*n* = 1), Sports and Recreation (*n* = 1), Development Planning (*n* = 1), and Urban Policy and Planning and group manager (*n* = 1).

**Table 2:** Details of participants from councils

Name of the councils	Number of interviews	Role of participants in local councils
Bankstown	3	Manager of Spatial Planning Community Safety and Crime Prevention Officer Team leader for Urban Policy and Planning open space planning
Camden	3	Strategic Planner Strategic Planner Team leader of Growth Areas
Liverpool	2	Manager of Strategic Planning Recreation and Open Space Planner
Campbelltown	4	Manager of Community Resources and Development Sport and Recreation Coordinator Strategic Planning Unit Development planner
Fairfield	2	Manager of city assets Group Manager, Governance and Community
Total	14	

### Themes

The results are grouped into two major themes, with the details of subthemes presented in Table 3. The presentation of the results is organized to describe [1] benefits and scope of engagement between councils and SWSLHD, and [2] proposed strategies and perceived enablers for effective engagement between councils and SWSLHD.

**Table 3:** Details of themes and number of references

Themes	No. of references
(1) Benefits and scope of engagement between councils and SWSLHD Subtheme 1: Insights into the benefits of engagement Subtheme 2: Scope of engagement of SWSLHD with councils	38 42
(2) Proposed strategies and perceived enablers for effective engagement between councils and SWSLHD Subtheme 1: Evidence on effectiveness of community-based programmes Subtheme 2: Improve communication between the council and SWSLHD	38 69

## Theme 1: Benefits and scope of engagement between SWSLHD and councils

This theme describes participants’ insight into the benefits of engagement with SWSLHD and their views on the scope of such an engagement.

### Insight into the benefits of engagement

This subtheme consists of a total of 35 references. Participants acknowledged that consultation with relevant stakeholders provides a better understanding of an issue they want to deal with and helps to generate different perspectives on proposed solutions. In addition, the consultation process helps to identify potential partners for the planning and implementation of proposed activities. Moreover, because of the nature of the partnership itself and the expertise of those involved, the activities are more likely to be evidence-based and with a greater potential to engage the community, thereby increasing the likelihood of enhancing the social and health outcomes.

*[Partnership provides] more of that human perspective…. We [from a council view] are looking at a patch of land rather than looking at an issue like health …[but] councils only exist for people… [and] sometimes that gets forgotten because we do have a very strong engineering focus; we have a very strong focus on plans, on maps and all that kind of thing … So, I think the local health district is important to bring back the people focus.*

### Scope of SWSLHD’s engagement with councils

This subtheme consists of a total of 42 references relating to the scope of engagement. Participants view the goals of the health sector and those of the council as having significant overlap, hence the need for consultation to discuss plans, strategies and activities, in order to achieve better health outcomes for residents.

*… if health had a different perspective and they had different ideas of what they wanted to do and they were contrary to what we were trying to do, it wouldn’t potentially work or may take some time to work out what to do; but currently there is a lot of overlap, like a serious amount of overlap.*

Further, participants agreed that while the main aim of councils is to design appropriate activities to engage community members, their activities also contribute to secondary outcomes such as social and health benefits that promote health.

*Our primary driver is not necessarily the health of the community; it’s more the engagement of the community in the activities they want to do. [Partnership with SWSLHD] certainly gives you other health benefits and it certainly gives you social benefits.*

Participants also considered that, as health is integrated into the work that councils do, there is a mutually advantageous opportunity for SWSLHD and councils to engage with each other. An example of this is councils’ activities around the built environment, such as designing and developing buildings, city centres, footpaths, gyms in parks, leisure centres, childcare centres. The existence of such facilities tends to encourage communities to become more physically active—such as walking, cycling and exercising in a variety of ways. These activities could potentially contribute to community-level health promotion, both in terms of the built environment and as a means of addressing the social determinants of health.

*We don’t deliver direct health services but we put gyms in parks; we run gym programmes, leisure centres, childcare centres, and everything we do touches on health both environmentally and in the social determinants or the risk-factor behaviour stuff as well.*

They consider collaboration between urban planners and health promotion service as essential, because:

*The urban design issue is a really important one, and then how that interacts with activity, so whether it’s sporting or just people being physically active or how easy it is for people to access transport, there are a lot of issues around urban design that are critical to us.*

So, within this context, participants suggested that it was imperative for councils to partner with SWSLHD in the design and delivery of appropriate activities.

## Theme 2: Proposed strategies and perceived enablers for effective engagement

This theme describes the council staff-perceived enablers that make possible an effective engagement between SWSLHD and councils. The required enablers discussed by participants included provision of evidence-based information on effectiveness of community based programmes, improvement in community engagement and effective communication between SWSLHD and councils. Equally important to the success of the planning process is the timing of the engagement with SWSLHD (and other relevant stakeholders).

### Evidence on effectiveness of community-based programmes

This subtheme consists of 38 references and presents the expectation of councils with respect to partnership with SWSLHD. Participants agreed that there was a dearth of evidence on various aspects of planning for community-based programmes and their health benefits, a gap which it is hoped the SWSLHD can help to fill. Councils have limited access to local data on effectiveness of smoking cessation programmes, the relationship between increased access to healthy food and physical environment and health outcomes, and access to well-being programmes that reduce risks from consumption of alcohol, gambling practices, and so on. Therefore, before they can deliver appropriate programmes, councils need clear and succinct evidence-based information about the local community, as emphasized by one participant:

*They (SWSLHD) need to do more research so we can do evidence-based planning, because, at the moment, we get very little local data. There is some. You can get some hospital data but we don’t get locally applicable data on smoking interventions [rates], data around increased access and engagement opportunities that increase physical activity; food environment and its association with improved consumption of fruits and vegetables and health outcomes. The current state or national data is not enough to help with our planning cycles.*

Participants noted that evidence-based information would also help the council to support its decisions in the situation of competing interests. For example, as one participant explained:

*One thing health could do would be to give us evidence on the social impact of gambling practices so that we can stand up. Councils approve applications for gambling [licenses] because we don’t have data to make decisions to stop them. Often, our hands are tied by the rules set by the state government.*

### Evidence on effectiveness of community engagement programmes

Participants also identified social engagement as an important area of work which requires evidence-based information if it is to be boosted, thereby increasing utilization of such things as sports programmes and recreation facilities. This relies on assistance from SWSLHD, as mentioned:

*Social engagement is the biggest problem, whether it be in sport, recreation or whatever, especially in some areas of local health district. So, helping us engage with the community and giving us more access to evidence are probably the two areas in most need of attention.*

### Improve communication between the council and SWSLHD

This subtheme consists of a total of 69 references from participants. The theme presents findings on how to improve communication between councils and SWSLHD, particularly as it relates to proactive cooperation with councils’ planning departments and developing stronger relationships overall.

### *Proactive liaising with council’s department of planning*:

Rather than waiting to provide input during the public notification and submission invitation periods, SWSLHD can proactively communicate with relevant council staff whenever they have cause, especially in the early stages of planning. Such involvement of relevant stakeholders is not only critical; it is also highly mutually beneficial.

*Where I see a space for you guys is, and that is from the submission perspective, which is one thing, but where I see a space is in terms of providing the evidence base early, so that, rather than waiting for submissions to be called, be proactive and think about the needs of a community. That means looking at our community strategic plan, and then looking at different actions *associated* with that and maybe providing the evidence and research that could help make a real difference on the ground.*

Hence, the SWSLHD needs to liaise with council planning departments, which may open even broader and deeper channels of engagement and connection with councils. Attending monthly council meetings including regional-level planning meetings; for example, meetings of the Western Sydney Regional Organisation of Councils could be another step towards understanding the nature of the work of councils, their requirements and how stakeholders can be part of the engagement process. Participants also suggested that, in addition to senior staff, the attendance and participation of lower- and middle-level staff at such meetings might also be useful.

As mentioned earlier, proactive engagement, apart from formal meetings, is critical to establishing an effective collaborative relationship, particularly one with a view to promoting community health:

*You need to have formal and informal meetings and you need it at an executive level or managerial level, but you also need it between.*

Participants indicated that in some situations, additional individual meetings with partners are organized to clarify issues of concern (emerging from initial consultations) to build consensus and working relationship. From time to time other options may also arise:

*There are often crossovers between, say, yourself and a different agency, or an issue someone else has that is going to impede being able to deliver what you want. So, we have that discussion and, later on, it’s often resolved through an individual meeting, and it’s easier to organize a meeting with one other than with 15 agencies.*

In summary, participants viewed that the feedback from stakeholders is assessed in terms of the value they, as partners, can add to the proposed plans or activities and whether they fit into the available resources. Needless to say, the feedback must be precise and evidence-based:

*I suppose the relevance, whether or not the concerns are being addressed, and whether it offers an objection or provides support, which is the key. We also have to look at how it fits with our policy decision-making to see whether there are synergies there.*

For SWSLHD to make a meaningful input, one participant advised specificity:

*So, if you are providing feedback on a review of a local environment plan for example, and I suppose we would benefit from evidence-based research, we would prefer less broad statements.*

### Stronger relationship

Participants acknowledged that both councils and SWSLHD need to work on building and maintaining stronger relationships to achieve effective outcomes from the formal consultation process. In this context, they urged having a contact person(s) in the SWSLHD who could be regularly engaged with councils, as well as easily contactable for information and updates, bypassing time consumption by bureaucratic processes:

*Probably the best way to do it would be to give us a contact officer within the department, someone we could go to and seek advice from the relevant authority, and someone who engages with us regularly.*

## Discussion

This study investigated opportunities for SWSLHD to engage with councils within its jurisdiction and identified approaches for more effective collaboration. The three main approaches discussed were as follows: providing local governments with evidence-based data to address issues of mutual concern that arise during the consultation process; integrating the goals of the SWSLHD with those of councils; and regular ongoing and proactive communication between SWSLHD and councils.

### *Providing evidence-based local-level data*:

The findings suggest that councils’ demand for evidence-based information on their communities has been largely unmet, a finding echoed in other studies [29, 30]. Our results concurred with those of recent research [3] which found a relative scarcity of good-quality evidence on the association between the provision of healthier and affordable food in diverse settings (e.g. supermarkets, schools, workplaces) and improved consumption of fruits and vegetables, or improved physical activity by establishing its correlation with increased access and engagement opportunities (e.g. increase infrastructure for walking and cycling).

Whereas national- or state-level data are available, there is a dearth of high-quality, local-government-level data that could be used to enable strategic decision-making, particularly in situations of competing budget priorities. For example, a recent systematic review by Bird et al. [3] identified a wealth of national- and global-level evidence on the association between improved food and physical environment and improved health outcomes (e.g. improved diet behaviours and weight-related outcomes). Such evidence, however, still needs to be tested on local populations and translated to the local government level as echoed in this study and a recent study [29].

One important implication of this study is in making the connection between upstream and downstream determinants of health. Research on the social determinants of health has advocated for more practical information on cross-sectoral policy decision-making processes, effective interventions and the impact of social determinants of health [11]. Furthermore, the available evidence base on the social determinants of health has been largely oriented towards downstream factors (behavioural interventions such as establishing parks and cycle paths and the associated impact on health outcomes) [19]. There is not only a lack of evidence for the effectiveness of interventions aimed at addressing upstream determinants of health; there are also challenges in applying evidence to understand what works for public health [31, 32]. Since local government impacts the structural social determinants of health through services and contribution to the creation of a healthy environment, it is critical that councils are provided with evidence of the effectiveness of such initiatives [31]. Therefore, within this context, this study strongly recommends that local health districts and councils collaborate to establish a local evidence base to demonstrate what works in interventions that address upstream determinants of health.

The study confirms the importance of providing locally relevant public health data to help address issues of mutual concern that arise during the consultation process. For example, high-quality evidence on how to improve community engagement of built physical environmental facilities (such as sports facilities and parks) would not only support councils in making complex, policy-focused decisions, but would also contribute to better engagement with council services. Hence, in consensus with other studies [29, 30], our findings advocate for local health districts to provide locally relevant health evidence to support planning and funding decisions of local governments.

### Integrating the goals of the SWSLHD with those of councils

The study found that coordination of relevant organizational-level policies and programmes between the SWSLHD and councils is vital for appropriate design and delivery of food and physical environmental plans and programmes of mutual concern. This notion has been found in previous studies, for example, in the evaluation of the Make Healthy Normal, an obesity prevention mass media campaign of the NSW government [33], and evaluation of the Healthy Eating Active Living programme of the Victorian Government through council-operated sports and recreation facilities [34]. Indeed, the number and variety of council partnerships with organizations representing education, transport, police and health is such that, where possible, the timely and effective leadership provided by local councils is critical towards achieving common goals.

### Regular ongoing and proactive communication

The study further found that maintaining an effective relationship between councils, SWSLHD and relevant other stakeholders from the inception of a project increased the likelihood of a successful preventive health effort. The results, consistent with the “five conditions of collective impact” proposed by Preskill et al. [35], highlight the (unmet) need for proactive and ongoing communication between the SWSLHD and councils. Such communication is crucial for multiple reasons: to understand a shared vision for change, to clarify issues of mutual concern, to collect evidence-based data reflecting the needs of councils, and to build consensus, maintain sustainable relationships and achieve collective impact.

In short, the study findings demonstrated that consultation with planning departments and participation in formal and informal periodic meetings with all levels of management is crucial and essential for understanding the nature of how councils operate and, importantly, how local health departments can be part of the engagement process. This is consistent with the recommendations of other studies, which suggested that local government is an underutilized resource in effective and efficient population health planning [12, 17, 30, 36]. Furthermore, federal government policies and guidelines typically fail to provide clear recognition of the value of collaboration with non-health sectors and instructions on how best to engage with local government [12]. Our study results confirmed that establishing an ongoing relationship between key managers and leaders of the local health district and their council counterparts is the most appropriate way to address communication gaps and increase the potential for effective collaboration. In this way, councils also gain a better understanding of the nature and scope of the local health district’s population health programmes [29].

The roles of local government in the promotion of public health across Australia are by and large similar [10], with the recognition that they have the potential to influence the social determinants of health and improve health equity gaps [12, 14, 15]. The concerns raised in this study about the need for practical information on effective interventions that impact on social determinants of health echo the findings from studies that were conducted in other local government settings in Victoria, South Australia and NSW [11, 12]. Hence, the findings from this study involving the staff of five of the seven councils in South Western Sydney could be generalized to other local government settings in NSW as well as other states of Australia.

The fact that data were collected from a single source, and not triangulated using other sources (e.g. consultations with political and other community-based stakeholders), is a major limitation of this study (although relevant policy papers were reviewed). On a more positive note, however, the findings were based on information gathered from local council staff working in areas directly related to policy, local environmental and community development. Another strength of the study is that perspectives were gathered from five of the seven councils.

In conclusion, this study found that there is a need to align the goals of local health districts with those of councils, a need to provide public health evidence with local applicability, particularly in the planning and consultation phase for issues of mutual concern. Additionally, proactive and ongoing consultations between local health districts and councils are recommended as a means of enabling effective engagement, and coordinated and sustained actions. Local health districts need to respond to these concerns regarding the need for more practical information on effective interventions and cross-sectoral decision-making processes while engaging with local government and other stakeholders.

## Data Availability

The qualitative data set which is stored in NVivo can be provided upon request.

## References

[1] World Health Organization. It's time to build a fairer, healthier world for everyone, everywhere. Health equity and its determinants. 2021.

[2] Marmot M, Friel S, Bell R, Houweling TA, Taylor S (2008). Closing the gap in a generation: health equity through action on the social determinants of health. Lancet (London, England).

[3] Bird EL, Ige JO, Pilkington P, Pinto A, Petrokofsky C, Burgess-Allen J (2018). Built and natural environment planning principles for promoting health: an umbrella review. BMC Public Health.

[4] McCormack GR, Shiell A (2011). In search of causality: a systematic review of the relationship between the built environment and physical activity among adults. Int J Behav Nutr Phys Act.

[5] Gomez LF, Sarmiento R, Ordoñez MF, Pardo CF, de Sá TH, Mallarino CH (1982). Urban environment interventions linked to the promotion of physical activity: a mixed methods study applied to the urban context of Latin America. Soc Sci Med.

[6] Gascon M, Triguero-Mas M, Martínez D, Dadvand P, Rojas-Rueda D, Plasència A (2016). Residential green spaces and mortality: a systematic review. Environ Int.

[7] Yen IH, Michael YL, Perdue L (2009). Neighborhood environment in studies of health of older adults: a systematic review. Am J Prev Med.

[8] D'Haese S, Vanwolleghem G, Hinckson E, De Bourdeaudhuij I, Deforche B, Van Dyck D (2015). Cross-continental comparison of the association between the physical environment and active transportation in children: a systematic review. Int J Behav Nutr Phys Act.

[9] Gibson M, Petticrew M, Bambra C, Sowden AJ, Wright KE, Whitehead M (2011). Housing and health inequalities: a synthesis of systematic reviews of interventions aimed at different pathways linking housing and health. Health Place.

[10] Parliament of New South Wales Government A. The Roles and Responsibilities of Federal, State and Local Governments. https://www.parliament.nsw.gov.au/about/pages/the-roles-and-responsibilities-of-federal-state-a.aspx. 2021. Accessed 13 May 21.

[11] Lawless A, Lane A, Lewis FA, Baum F, Harris P (2017). Social determinants of health and local government: understanding and uptake of ideas in two Australian states. Aust N Z J Public Health.

[12] Javanparast S, Baum F, Freeman T, Ziersch A, Henderson J, Mackean T (2019). Collaborative population health planning between Australian primary health care organisations and local government: lost opportunity. Aust N Z J Public Health.

[13] Jolley G, Barton E (2015). Local government capacity to deliver health promotion initiatives: a case study. Health Prom J Austr.

[14] World Health Organization Regional Office for Europe. Addressing the Social Determinants of Health: The Urban Dimension and the Role of Local Government. Liège (BEL): WHO Regional Office for Europe; 2011.

[15] World Health Organization. Commission on Social Determinants of Health. Closing the Gap in A Generation: Health Equity Through Action on the Social Determinants of Health. Geneva (CHE): World Health Organization; 2008. 10.1016/S0140-6736(08)61690-6 18994664

[16] VIC Department of Human Services. Environments forHealth. Promoting Health and Wellbeing through Built, Social, Economic and Natural Environments. Municipal Public Health Planning Framework. Melbourne (AUST): State Government of Victoria; 2001. Accessed on 14.07.21: https://www2.health.vic.gov.au/about/publications/researchandreports/Environments-for-Health-Municipal-Public-Health-Planning-Framework. 2001.

[17] NSW Ministry of Health. Healthy Built Environment Checklist. A guide for considering health in development policies, plans and proposals. Sydney: NSW Department of Health. Accessed on 20 April 2021. https://www.health.nsw.gov.au/urbanhealth/Pages/healthy-built-enviro-check.aspx. 2020.

[18] Marmot M (2010). The Marmot Review: Fair Society, Healthy Lives – Strategic Review of Health Inequalities in England post-2010.

[19] Phillips G, Green J (2015). Working for the public health: politics, localism and epistemologies of practice. Sociol Health Illn.

[20] Collins PA (2012). Do great local minds think alike? Comparing perceptions of the social determinants of health between non-profit and governmental actors in two Canadian cities. Health Educ Res.

[21] NSW Government Health. Governance and legislation for local health district and specialty network boards NSW: NSW Health; 2020. https://www.health.nsw.gov.au/lhd/boards/Pages/board_governance.aspx.

[22] Chok HN, Thornell M, Maxwell M, Wise M, Sainsbury P (2014). Population health services can influence land use planning. Aust N Z J Public Health.

[23] Janzen C, Marko J, Schwandt M (2018). Embedding health equity strategically within built environments. Can J Public Health..

[24] Tong A, Sainsbury P, Craig J (2007). Consolidated criteria for reporting qualitative research (COREQ): a 32-item checklist for interviews and focus groups. Int J Qual Health Care.

[25] NSW Government H, South Western Sydney Local Health District. Growing Healthy Kids in South West Sydney: SWSLHD Childhood Overweight and Obesity Prevention and Magaement Action Plan 2017–2025 2017.

[26] South Western Sydney Local Health District. South Western Sydney Local Health District-Year in review for 2019–2020. 2020.

[27] Braun Virginia CV (2006). Using thematic analysis in psychology. Qual Res Psychol.

[28] Castleberry A (2014). NVivo 10 [software program] Version 10 QSR International. Am J Pharm Educ..

[29] Kneale D, Rojas-García A, Thomas J (2019). Obstacles and opportunities to using research evidence in local public health decision-making in England. Health Res Pol Syst.

[30] Pettman TL, Armstrong R, Pollard B, Evans R, Stirrat A, Scott I (2013). Using evidence in health promotion in local government: contextual realities and opportunities. Health Prom J Austr.

[31] Barton H, Grant M (2013). Urban planning for healthy cities. A review of the progress of the European Healthy Cities Programme. J Urban Health..

[32] Kelly M, Morgan A, Ellis S, Younger T, Huntley J, Swann C (2010). Evidence based public health: A review of the experience of the National Institute of Health and Clinical Excellence (NICE) of developing public health guidance in England. Soc Sci Med..

[33] Kite J, Thomas M, Grunseit A, Li V, Bellew W, Bauman A (2020). Results of a mixed methods evaluation of the Make Healthy Normal campaign. Health Educ Res.

[34] Office of Local Government-New South Wales of Australia. Community Engagement Strategy. https://www.olg.nsw.gov.au/councils/integrated-planning-and-reporting/framework/community-engagement-strategy. Accessed 13 May 21.

[35] Preskill H, Parkhurst, Marcie, Splansky Juster, Jennifer,. The Guide to Evaluating Collective Impact by FSG. https://paulallen.ca/documents/2015/10/preskill-h-parkhurst-m-and-juster-js-learning-and-evaluation-in-the-collective-impact-context-2014.pdf/. 2014. . Accessed 30 Apr 21

[36] Riesenberg D, Blake MR, Boelsen-Robinson T, Peeters A, Cameron AJ (2020). Policies influencing the provision of healthy food and drinks in local government-owned sport and recreation facilities in Victoria, Australia. Aust N Z J Public Health.

